# The effectiveness of biosecurity interventions in reducing the transmission of bacteria from livestock to humans at the farm level: A systematic literature review

**DOI:** 10.1111/zph.12807

**Published:** 2021-02-04

**Authors:** Dina Mohamed Youssef, Barbara Wieland, Gwenan M. Knight, Jo Lines, Nichola R. Naylor

**Affiliations:** ^1^ London School of Hygiene & Tropical Medicine (LSHTM) London UK; ^2^ International Livestock Research Institute (ILRI) Addis Ababa Ethiopia

**Keywords:** bacterial infections, biosecurity interventions, effectiveness, farm level, livestock, occupational risk

## Abstract

Zoonotic bacterial infections are a health hazard for people who are in regular contact with livestock at the farm level. Improved biosecurity can limit zoonotic pathogen transmission within farms. The aim of this review was to summarize the effectiveness of farm‐level biosecurity interventions in reducing bacterial transmission from animals to people who lived, worked in or visited farms. A systematic literature review was conducted using Embase, Ovid Medline and Agris databases, which were searched on 7^th^ of July 2019, limited to English language papers but with no time exclusion criteria. A narrative synthesis was undertaken utilizing the Centre for Reviews and Dissemination approach, reported in accordance with Preferred Reporting Items for Systematic Reviews and Meta‐Analyses guidelines. Risk of bias within and across the included studies was performed using established checklists. Out of 869 studies retrieved through database searches, 11 studies were selected. In addition, three studies were found through study reference lists. Fourteen studies were therefore included in this review. Biosecurity interventions were grouped into five categories: hand washing, sanitization and hygienic measures (six studies); personal protective equipment (five studies); vaccination (two studies); other interventions (e.g. air ventilation flap) (four studies); and routine farm activities (two studies). Across studies that investigated odds of human colonization or infection (three studies), odds were seen to both be increased and decreased through use of tested biosecurity measures. Large confidence intervals that often crossed the threshold of an odds ratio equal to 1 were found. Most of the studies' overall risk of bias was ‘medium risk’ (11 studies), with selection bias domains generally being scored ‘medium risk.’ Biosecurity interventions are potentially beneficial in reducing bacterial transmission from animals to humans. However, more high‐quality evidence is needed to increase certainty in which interventions, in which contexts, are most effective from the human health perspective.

**Impact**

- Understanding the effectiveness of farm‐level biosecurity interventions from a human health perspective allows farmers and policymakers to select the most effective biosecurity measures, not only in terms of animal health but also in terms of public health.
- The majority of studies that were found estimated the impact of interventions directly targeting the human–animal interface (including hygiene and personal protective equipment), and were based in Europe.
- Across all intervention groups, there was evidence of reduced human bacterial colonization and/or infection outcomes, but alongside high uncertainty in effect size and direction in studies investigating odds ratios.

## 1 INTRODUCTION

### 1.1 Rationale

Zoonotic bacterial infections are a serious public health hazard, as well as an occupational risk for farm workers who are in regular contact with livestock. Previous research indicates that there is transmission of methicillin‐resistant *Staphylococcus aureus* (MRSA) from animals to farmers or those in direct contact with livestock (Graveland et al., 2011; Pletinckx et al., 2013; Van den Broek et al., 2009). In addition, risk of infections from microbes such as *Campylobacter* and *Brucella* species has been found to be increased in people who are in direct contact with animals (El‐Tras et al., 2015; Te‐Chaniyom et al., 2016). Increased zoonotic infections, especially antibiotic resistant ones such as MRSA, may increase the burden on healthcare systems and reduce agricultural labour force productivity (Grace et al., 2012). Therefore, understanding how to reduce their emergence and transmission is important from a public health and economic perspective.

Biosecurity is defined by the Irish Department of Agriculture, Environment and Rural Affairs as ‘The prevention of disease causing agents entering or leaving any place where they can pose a risk to farm animals, other animals, humans, or the safety and quality of a food product’ (Department of Agriculture, Environment, & Rural Affairs, 2019). Improved biosecurity is an often‐applied intervention to limit pathogen transmission within and between farms. These interventions can also affect transmission of zoonotic pathogens to humans, thus having a beneficial impact on public health. Types of biosecurity interventions and their effectiveness to reduce transmission of bacterial infections from animal to farmers are available (Schimmer et al., 2012). However, the effectiveness of these interventions in terms of the reduced transmission of bacteria to humans has not been systematically analysed or assessed in terms of risk of bias. Understanding the effectiveness of such interventions, alongside the strength of evidence, is important for selecting efficient interventions from a One Health perspective.

### 1.2 Aim and objectives

The aim of this review was to summarize the effectiveness of biosecurity interventions in reducing the transmission of bacterial infections from livestock to humans at the farm level.

To meet the aim of this review, the following objectives were set:

1. To collate and describe evidence that assesses the effectiveness of biosecurity interventions in reducing bacterial transmission from animals to people at the farm level. Effectiveness was defined in terms of bacterial (colonization or infection) transmission, prevalence, incidence, intervention cost‐effectiveness and/or cost benefit.
2. To assess the risk of bias within and across the current evidence on the effectiveness of biosecurity interventions on the transmission of bacteria from animals to people, within farms.

## 2 METHODS

### 2.1 Study design

This is a systematic review guided by the Center for Reviews and Dissemination (CRD) report on conducting systematic reviews (Centre for Reviews & Dissemination, 2009), in particular applying its narrative synthesis framework. Reporting is in line with the Preferred Reporting Items for Systematic Reviews and Meta‐Analyses (PRISMA) checklist (Moher et al., 2009) (see Appendix S1 Table 1.1 for the completed checklist).

### 2.2 Eligibility criteria

The inclusion and exclusion criteria (Table 1) of selected studies in this review were based on the Population, Intervention, Comparison, Outcome, and Study design (PICOS) framework (Centre for Reviews & Dissemination, 2009).

**TABLE 1:** Inclusion/exclusion criteria applied using the ‘Population, Intervention, Comparison, Outcome, and Study design (PICOS)’ framework

Category	Inclusion criteria	Exclusion criteria
Population	Populations of interest were livestock and people^a^. People included farm workers and professionals, visitors or people in contact with livestock in the farm	Studies including only people or only livestock^a^ Fish and crop farms
Interventions	Biosecurity interventions Farm activities that seem to impact bacterial infections in people and acting as a biosecurity	Interventions limiting foodborne infection transmission only
Comparison	Biosecurity interventions compared with each other or with no intervention	
Outcome	Effectiveness of interventions in terms of bacterial infections, colonization, prevalence, incidence, risk and relative risk measures related to human health Economic evaluation measures including cost‐effectiveness and cost‐benefit associated with human health outcomes	The outcome of interventions limiting spread of infections amongst animals only Effectiveness of interventions on microbes other than bacteria only The cost of intervention implementation without a cost of outcome Studies stating only the interventions without an impact outcome
Study design	All analytical study designs for biosecurity interventions impact such as cohort, case control and cross‐sectional, in addition to experimental studies Descriptive cross‐sectional studies Economic evaluation studies for cost‐effectiveness and cost benefit	In vitro intervention studies. Qualitative research study design Descriptive studies such as level of intervention prevalence or intervention implementation without outcomes of their impact
Other	English language studies	Conference abstracts

^a^ Livestock was included according to the livestock definition mentioned by the United Kingdom's Department of Environment, Food and Rural Affairs (Department of Environment, Food, & Rural Affairs, 2011).

### 2.3 Search methods

#### 2.3.1 Information sources

Embase (Embase classic + Embase: from 1947 to 7 July 2019) (Ovid Embase, 2019), Ovid Medline (from 1946 to 7 July 2019) (Ovid Medline, 2019) and Agris (Food & Agriculture Organization of the United Nations, 2019) databases were searched on 7 July 2019.

#### 2.3.2 Search strategy

There were five concepts of search terms used in the search strategy: biosecurity, interventions, livestock, farm workers and effectiveness. A comprehensive list of relevant synonyms and mesh terms (subheadings) was used for each term. Boolean operators such as AND and OR were used to build the search. In addition, truncation signs and wild cards were used to broaden the search. All search terms used were consulted with an information specialist of London School of Hygiene and Tropical Medicine library for any recommendations. Search terms for AGRIS database were (Biosecurity) AND (Intervention* OR polic* OR measure* OR strateg* OR methods OR procedures OR techniques) AND (Farmer OR farm* OR farm‐level OR livestock OR "Farm animal*") AND (Effectiveness OR incidence OR prevalence OR cost‐effectiveness OR cost‐benefit OR outcome* OR "bacterial infection" OR "bacterial colonization" OR "bacterial colonisation" OR colonization OR colonisation OR "microbial colonization" OR "microbial colonisation" OR "cost‐effectiveness analysis" OR "cost‐effectiveness ratio" OR "cost‐benefit ratio" OR "cost‐benefit analysis" OR "cost analysis"). A full list of search terms for each database used can be found in Appendix S2.

English language filters were utilized. No search limits were applied on publication dates or quality of studies. Relevant references from the selected studies were identified, consulted and included in the review if suitable.

### 2.4 Study selection

All studies were exported from Web interfaces of the databases into an Excel sheet using the ‘CSV’ options. Removal of duplicates was conducted using the Microsoft Excel ‘Remove Duplicates’ function (Microsoft Corporation, 2010) based on author and title, then manually for any remaining duplicates. The inclusion and exclusion criteria (Table 1) were then applied at two stages (‘title and abstract’ screening, then ‘full‐text’ reviewing) by two independent reviewers. The first reviewer reviewed 100% of the retrievals at each stage. The second reviewer reviewed 25% for the ‘title and abstract’ stage, and 27% for the ‘full‐text stage.’ This is due to articles that could not be accessed being re‐reviewed through titles and abstracts, changing the denominator of full texts. Agreement at the first stage was (81.75%). Studies in which disagreement occurred were discussed. It was agreed that all studies that at least one reviewer had included should enter the next stage of review to reduce the likelihood of wrong exclusion. Agreement was (100%) for the full‐text stage.

### 2.5 Data collection process

A data extraction table was constructed by the first reviewer. It was then refined and agreed upon between the first and second reviewer. It was developed in Microsoft Excel (Microsoft Corporation, 2010), with data items as described in Section 2.6. The first reviewer extracted the data from the included studies. Any studies with unclear results (as determined by the first reviewer) were checked by the second reviewer (four out of 14 studies).

### 2.6 Data items

In line with the agreed data extraction table, the following variables were extracted: study reference (title and author), study aim, study location, population, type of infection, intervention type, study's risk of bias, outcome measures, study designs and results. All relevant results were extracted, including those from univariate analyses. If a study had multiple results for different interventions (or different categories within those interventions), all results were extracted.

### 2.7 Risk of bias in individual studies

Risk of bias assessment of the included studies was conducted using the National Institute of Health tool for cross‐sectional, cohort, case–control and pre–post study designs (National Heart, Lung, & Blood Institute, 2019). For a cross‐sectional descriptive study, the tool was utilized with certain items scored as ‘not applicable.’ The Critical Appraisal Skills Programme (CASP) tool was used for economic evaluation studies (Critical Appraisal Skills Programme, 2018).

Risk of bias was assessed for each study according to the presence of selection bias, information bias (measurement bias) and confounding as explained by the tool guidance (National Heart, Lung, & Blood Institute, 2019). For each criterion of bias, a scoring of low, medium or high risk was given according to how many biases were found for the same criteria (see Appendix S6). For example, if two types of selection bias were assessed (such as responder bias and selection of participants from different populations), studies that had only one type were scored as ‘medium risk of selection bias’ and those with more than one type were scored as ‘high risk of selection bias.’ A high‐risk score was also given for studies with no control of confounding.

This review assessed overall study risk of bias as:

1. A study including two or more high risk of bias indicators was considered to have an overall high risk of bias.
2. A study including one or more medium risk of bias indicators was considered to have an overall medium risk of bias.
3. A study including two low‐risk and one high‐risk indicators was considered as overall medium risk.
4. A study including only low risk of bias indicators was considered to have an overall low risk of bias.

The CASP tool had no scoring system; however, the appraisal depended on three main questions, which were addressed whilst completing the checklist for economic evaluation studies: (a) Is the economic evaluation valid? (b) How were consequences and costs assessed and compared? and (c) Will the results help in purchasing for local people? (Critical Appraisal Skills Programme, 2018).

### 2.8 Summary measures

The primary outcome was the reduction of bacterial infection/colonization transmission from animal to people with odds ratios (ORs), relative risks (RRs), prevalence, colonization of the infection as principal summary effect measures. In addition, the cost‐effectiveness ratio and cost‐benefit ratio of the intervention in reducing bacterial infection transmission were used as summary effect measures.

### 2.9 Synthesis of results

Data were combined and compared using a narrative synthesis approach, in line with the CRD guidance (Centre for Reviews & Dissemination, 2009). No meta‐analysis was conducted due to heterogeneity in bacteria and measures of impact used in each study. First, an initial description of the studies' characteristics was done, alongside a preliminary synthesis of the results of the included studies. Studies were grouped by intervention type, and results were tabulated for visual presentation. Interventions were considered protective or not based on the stated effect sizes. For OR and RR, ORs < 1 and RRs < 1 were interpreted as protective effects, whilst ORs > 1 and RRs > 1 were interpreted as harmful effects. For prevalence measures (such as proportions), a decrease in prevalence of the outcome was interpreted as protective. *p*‐values (*p* < .05) and 95% confidence intervals (CIs) were used to describe statistical significance. As the narrative synthesis is an iterative process (Centre for Reviews & Dissemination, 2009), a theory of change linking the discussed biosecurity interventions to the outcomes of interest was then drawn out. To explore the relationship within and between studies, forest plots of available ORs were created, grouped by intervention type. No singular summary measure per intervention was given, as a meta‐analysis was not performed due to study characteristic heterogeneity. In addition, studies for interventions and infection types were compared per type of farm.

### 2.10 Risk of bias across studies

Risk of bias across studies was assessed through grouping studies by intervention type and outcome type (Cochrane, 2011). Across each of these groups, general trends were highlighted in overall study bias and individual criterion of bias.

## 3 RESULTS

### 3.1 Study selection

**FIGURE 1:**
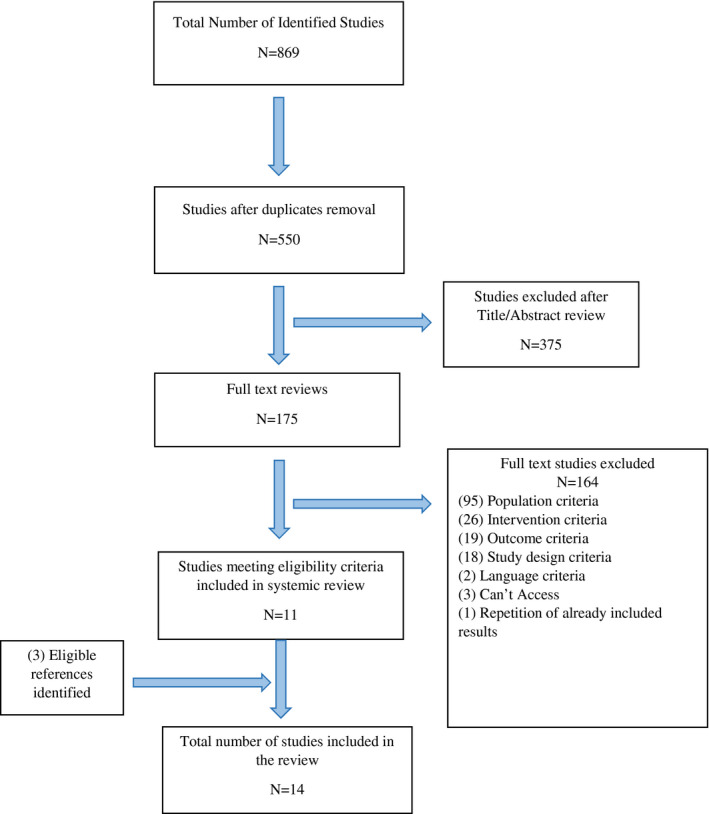
Preferred Reporting Items for Systematic Reviews and Meta‐Analyses flow diagram

Initially, 869 studies were identified from the three databases. After deduplication, 550 unique studies were retrieved, which were then screened by title and abstracts. Three hundred seventy‐five studies were excluded after title/abstract screening, and 175 studies were full text reviewed (Figure 1). Of these, 11 studies were selected to be included. In addition, three eligible references were identified from reference lists of these studies. Therefore, a total of 14 studies were included in this review.

### 3.2 Study characteristics

Study characteristics such as year, aims and objectives for each study are represented in Table 3.1 in Appendix S3.

The study locations were classified according to World Bank regional groupings (The World Bank, 2019). From the Europe and central Asia region, two studies were from the United Kingdom (UK) (Ridley et al., 2011; Williams et al., 2013), two from the Netherlands (Schimmer et al., 2012, 2014) and one from Belgium (Pletinckx et al., 2013). Whilst for the Middle East and North Africa region, two studies were from Egypt (Elmonir et al., 2019; El‐Tras et al., 2015). For the Asia and the Pacific region, one study from Thailand (Te‐Chaniyom et al., 2016), one from Australia (Bond et al., 2016) and one from Mongolia (Zinsstag et al., 2007) were identified. For the North America region, two studies were from Canada (Meadows et al., 2016; Racicot et al., 2013) and one was from the United States of America (USA) (Leedom Larson et al., 2010). Whilst for Sub‐Saharan Africa, one study was from Southern Ethiopia (Abdi et al., 2017). All studies' results are represented in Table 4.1 Appendix S4.

The most represented types of farms were poultry farms, with five studies (Abdi et al., 2017; El‐Tras et al., 2015; Racicot et al., 2013; Ridley et al., 2011; Williams et al., 2013). In addition, four studies were conducted in goat farms (Bond et al., 2016; Meadows et al., 2016; Schimmer et al., 2012; Te‐Chaniyom et al., 2016). Three studies were conducted in dairy cattle and cow farms (Elmonir et al., 2019; Schimmer et al., 2014; Zinsstag et al., 2007), where one of them included small ruminants and cattle (Zinsstag et al., 2007). Furthermore, two studies were conducted in pig farms (Leedom Larson et al., 2010; Pletinckx et al., 2013).

There were various bacterial infections identified in the included studies with the majority being *Coxiella burnetii* (*C. burnetii*; four studies) (Bond et al., 2016; Meadows et al., 2016; Schimmer et al., 2012, 2014). There were two studies each for *Salmonella* (Abdi et al., 2017; Racicot et al., 2013), MRSA (Leedom Larson et al., 2010; Pletinckx et al., 2013), *Brucella* (Te‐Chaniyom et al., 2016; Zinsstag et al., 2007) and *Campylobacter* (El‐Tras et al., 2015; Ridley et al., 2011). Also, one study each for *Escherichia coli* (*E. coli*) (Racicot et al., 2013), *Chlamydia psittaci* (Williams et al., 2013) and *Staphylococcus aureus* (*S. aureus*) (Elmonir et al., 2019).

Nine cross‐sectional studies, one cohort study, one case–control study and two pre–post studies were included. Additionally, one economic evaluation study was included.

### 3.3 Risk of bias within studies

Out of the 14 studies included in this review, 13 used the National Institute of Health risk of bias tool, whilst one study was assessed using CASP tool for economic evaluation study design (Zinsstag et al., 2007). Please refer to Appendix S5 for individual criterion of bias and overall risk of bias, and Appendix S6 for individual study risk of bias checklist results.

### 3.4 Results of individual studies

Among the 14 studies reviewed, five main types of biosecurity interventions were identified, which were as follows: (a) hand washing, sanitization and hygienic measures; (b) personal protective equipment (PPE); (c) vaccination; (d) other interventions (e.g. air ventilation flaps); and (e) changes to routine farm activities (e.g. changes to farmer practice when doing routine tasks that may affect bacterial transmission such as performing extended lactation). The most commonly studied biosecurity interventions were hand washing, sanitization and hygienic measures with six studies (Abdi et al., 2017; El‐Tras et al., 2015; Leedom Larson et al., 2010; Meadows et al., 2016; Racicot et al., 2013; Ridley et al., 2011). PPE interventions were found in five studies (Elmonir et al., 2019; Meadows et al., 2016; Schimmer et al., 2012, 2014; Williams et al., 2013). Vaccination was evaluated in two studies (Bond et al., 2016; Zinsstag et al., 2007). Other interventions were identified in four studies (Bond et al., 2016; Pletinckx et al., 2013; Schimmer et al., 2012, 2014), these included (e.g. high‐efficiency particulate arrestance [HEPA] filter and using automatic milking). Changes to routine farm activities were identified in two studies (Schimmer et al., 2012; Te‐Chaniyom et al., 2016). Appendix S4 provides a detailed table with individual study results.

#### 3.4.1 Hand washing, sanitization and hygienic measures

Bacterial count, prevalence and cases were found to be significantly lower from hand washing in studies based in Canada (Racicot et al., 2013) and the UK (Ridley et al., 2011). One Canadian study considered four different protocols, where all had a statistically significant, positive effect on initial hand contamination for total coliform count and total aerobic bacterial count with *p* < .0001 (Racicot et al., 2013). All hands washed were found negative for *Salmonella* with each of the protocols tested (Racicot et al., 2013). One UK‐based study found a reduction of *Campylobacter* prevalence from 14% to 10% in the hands of poultry catching members was found to be significantly associated with hand washing, *p* = .002 (Ridley et al., 2011), whilst another poultry‐based study found a 33% prevalence of *Salmonella* in hand swabs from farm attendants. Poor hand washing practices by attendants were considered as a risk factor for *Salmonella* prevalence (Abdi et al., 2017).

On the other hand, a different Canadian study did not find significant effects of hand washing on reducing *C. burnetii* infection/colonization (*p* = .19) (Meadows et al., 2016). This paper found results that were intuitively hard to accept, with ‘frequent and infrequent hand‐washing behaviour’ and ‘always hand washing only after assisting with normal birth’ being shown as having a positive odds of *C. burnetii* seropositivity, with very wide CIs indicating uncertainty. However, these factors were not explored in the multivariate analysis (see Table 4.1 Appendix S4 for full results) (Meadows et al., 2016).

Other hygienic measures were found to reduce the prevalence of bacterial infections. In poultry farms, poor cleaning and disinfection was found to be associated with a higher prevalence of *Campylobacter coli* and *Campylobacter jejuni* positive infections in households (the households term included both poultry and children based in the same household; *p* < .05), in Egypt (El‐Tras et al., 2015). In the UK, a reduction of *Campylobacter* prevalence from 41% to 19% in the footwear of poultry catching members was found to be significantly associated with footwear disinfection (*p* = .002) (Ridley et al., 2011). Whilst, in the USA, a study found that work laundry separation was found to be a possible risk factor for MRSA infection prevalence in pork producers, however this result was not statistically significant (*p* = .11) (Leedom Larson et al., 2010).

#### 3.4.2 Personal protective equipment

In relation to gloves, two studies were found. Meadows et al. estimated that farm workers who always wear gloves whilst assisting with presumed abortion had 0.37 times the odds of *C. burnetii* infection compared to farm workers never wearing gloves, which was a protective measure with significant evidence of negative association (OR: 0.37, 95% CI [0.072–1.92], *p* = .024). However, infrequent gloves wearing whilst assisting with presumed abortion was insignificant protective (OR 0.28, 95% CI [0.016–4.84], *p* = .38). Results relating to ‘Frequent gloves wearing’ were intuitively hard to accept, as being shown as having a positive odds of *C. burnetii* seropositivity compared to not wearing gloves, though all of these results are based on univariate analysis (Meadows et al., 2016). Another study showed that farm workers, in the Netherlands, who were fully compliant with gloves usage during cattle birth care were less likely to have *C. burnetii* infections compared to those partially or non‐compliant with gloves usage (OR: 0.4, 95% CI [0.2–0.8], *p* < .1) in a multivariate analysis (Schimmer et al., 2014).

With regard to the effectiveness of clothes and boots, *C. burnetii* infections in farm workers were evaluated in three studies. Farm workers always changing their clothes after assisting with birth stated as significantly less likely to get *C. burnetii* infection compared to farm workers never change their clothes (OR: 0.14, 95% CI [0.03–0.80, *p* = .027]) (Meadows et al., 2016). Whilst, professional farm visitors wearing boots and clothes had lower odds of *C. burnetii* infection compared to visitors not wearing boots and clothes (OR: 0.7, 95% CI [0.4–1.1], *p* < .2) in univariate analysis conducted in the Netherlands (Schimmer et al., 2014). In a multilevel analysis, another study in the Netherlands showed that farmers not using boots had higher odds of *C. burnetii* infection compared to those using boots [OR: 2.66, 95% CI [1.12–6.32], *p* = .025) (Schimmer et al., 2012).

The effectiveness of respiratory mask and eye protection was assessed as effect modifiers in one study in the UK in a poultry plant. This PPE did not statistically significantly modify the impact of working in ‘high‐risk’ areas on infection odds. However, it did significantly modify the impact of touching of the face with potentially infectious material (e.g. blood) (Williams et al., 2013).

One study found that farm workers had an overall prevalence of *S. aureus* of 88.9% in nostril swabs and 100% in hand swabs. The authors concluded since no farm workers were using PPE, this was a risk factor; however, no association between them was assessed (Elmonir et al., 2019).

#### 3.4.3 Vaccination

Two studies investigated vaccination impacts: one effectiveness and one cost‐effectiveness study (Bond et al., 2016; Zinsstag et al., 2007).

In a Q fever outbreak in a goat farm in Australia, human Q fever vaccine (Q‐Vax®, CSL Ltd) protection was evaluated and found to have 90% efficacy for farm workers vaccinated 15 days before exposure to the infection (in terms of vaccine efficacy) (Bond et al., 2016). An economic study evaluated the human benefits of animal interventions in Mongolia. The study evaluated the cost‐effectiveness of *Brucella melitensis* Rev‐1 for small ruminants and *Brucella abortus* S19 for cattle as annual mass vaccination for 10 years and found that achieving a reduction of brucellosis transmission by 52% between animals result in a human impact of avoiding 51,856 human brucellosis cases, which results in 49,027 disability‐adjusted life years (DALYs) averted, with a cost‐effectiveness ratio of US$19.1 per DALY averted (95% CI: 5.3–486.8) (Zinsstag et al., 2007).

#### 3.4.4 Other specific interventions

Three studies investigated interventions linked to air flows and filtration (Bond et al., 2016; Pletinckx et al., 2013; Schimmer et al., 2012). A HEPA filter was assessed in an Australian goat farm (Bond et al., 2016). The risk of *C. burnetii* infection among administrative staff in unfiltered adjoining offices and among workers regularly handling goat and kids was around five times the risk of infection among workers in a HEPA‐filtered factory (Bond et al., 2016). Another study showed that dairy goat farmers using air ventilation flaps had lower odds of *C. burnetii* infection compared to those not using air ventilation flaps in univariate analysis (OR: 0.52, 95% CI: [0.5–1.11]); however, evidence of association was deemed not statistically significant (*p* = .10) (Schimmer et al., 2012). In Belgium, a study looking at pigs, mixed broiler‐pig farms and mixed dairy‐pig farms found that a pig farm with separate buildings and separate air flow seemed to have lower prevalence of MRSA in farmers compared to farms without, though formal associations were not tested (Pletinckx et al., 2013).

Farmers using screen/gauze in stables and those using windstoppers only had high odds of *C. burnetii* infections (OR: 1.86, 95% CI [0.91–3.80] and OR 1.01, 95% CI [0.52–1.98], respectively) compared to using none of the above (Schimmer et al., 2012). Also, feeding method was assessed and found that farmers using fodder mixer/automatic had slightly higher odds of *C. burnetii* infection compared to those using hand/wheelbarrow methods (OR: 1.8, 95% CI [1.04–3.15] *p* = .04) (Schimmer et al., 2012). In addition, using automatic milking was found to be protective against *C. burnetii* in dairy cattle farm with lower odds of infection for farm workers compared to those not using automatic milking (OR: 0.7, 95% CI [0.4–1.0], *p* < .1) (Schimmer et al., 2014).

#### 3.4.5 Routine farm activities

This type of intervention included changes to routine farmer practices that may affect bacterial transmission and thus could act as a biosecurity measure. In the Netherlands, dairy goat farmers' odds of *C. burnetii* infection was estimated to be significantly associated with some farm activities (Schimmer et al., 2012). This included the use of silage, use of maize, milking goats, feeding goats, supply and removal of dairy goats or bucks, care for general animal health, birth assistance, spread manure and clean the stables (*p* < .01), use of artificial insemination (*p* = .04) and remove manure (*p* = .01). Farmers not performing extended lactation had lower odds of infection compared to those performing extended lactation (OR: 0.37, 95% CI [0.15–0.86], *p* = .036) (Schimmer et al., 2012). A study in Thailand found that the *Brucella* prevalence of livestock officers was 8.8% (95% CI [1.9–23.7]), but was not significantly associated with any of their performed tasks on goat farms such conducting vaccination, contacting placenta and vaginal secretions, blood collection, artificial insemination (Te‐Chaniyom et al., 2016).

### 3.5 Synthesis of results

**FIGURE 2:**
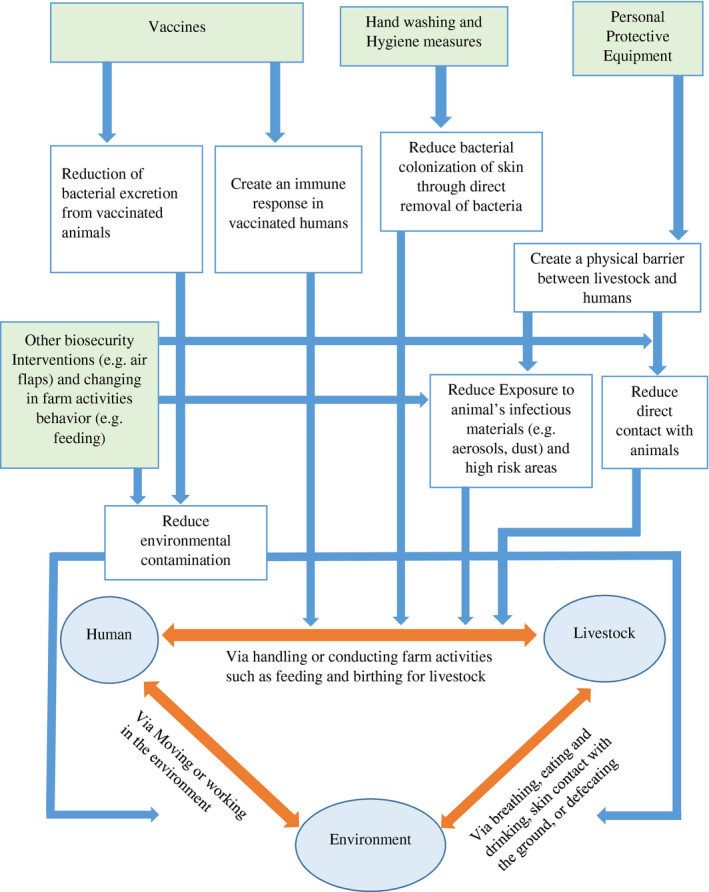
Implicit theory of change for included biosecurity interventions groups in reducing the transmission of bacteria between livestock and humans at the farm level. Circles represent reservoirs, orange arrows represent potential routes of bacterial transmission, green boxes represent interventions and white boxes represent possible effects of interventions.

The initial theory of change for farm‐level biosecurity interventions in potentially changing transmission of bacteria between livestock and humans (Figure 2) was built. This was based on the interventions found from the literature, depicting their links to each other through their connection to the animal–human–environment interface of bacterial transmission routes. This shows that whilst most of the intervention groups target the direct transmission across humans and livestock (through physical barriers or direct removal of bacteria), other interventions discussed in this review also target transmission indirectly through the environment.

**FIGURE 3:**
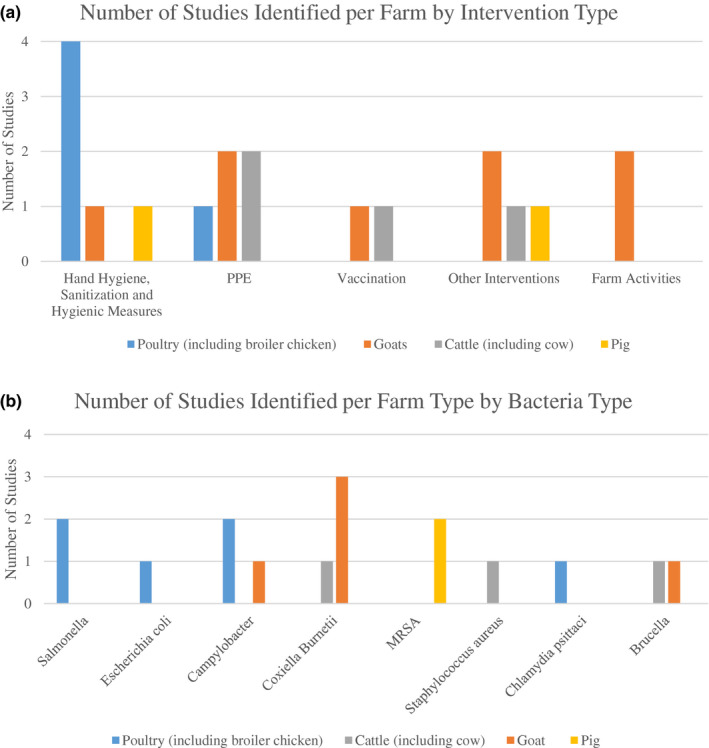
Number of studies identified per farm type by (a) intervention type and (b) bacteria

Poultry farms were the most common type of farms where hand hygiene, sanitization and hygienic interventions were identified (Figure 3a), whilst cattle and goat farms were the most common types of farms where PPE interventions were identified. Farm activity‐related results were only identified in goat farms. In addition, all of the studies related to *Salmonella* were found to be in poultry farms (Figure 3b), whilst all of the studies related to MRSA were in pig farms. Three out of the four studies, which were done on *C. burnetii* infections, were in goat farms. The only Gram‐positive bacteria investigated were *S. aureus*.

**FIGURE 4:**
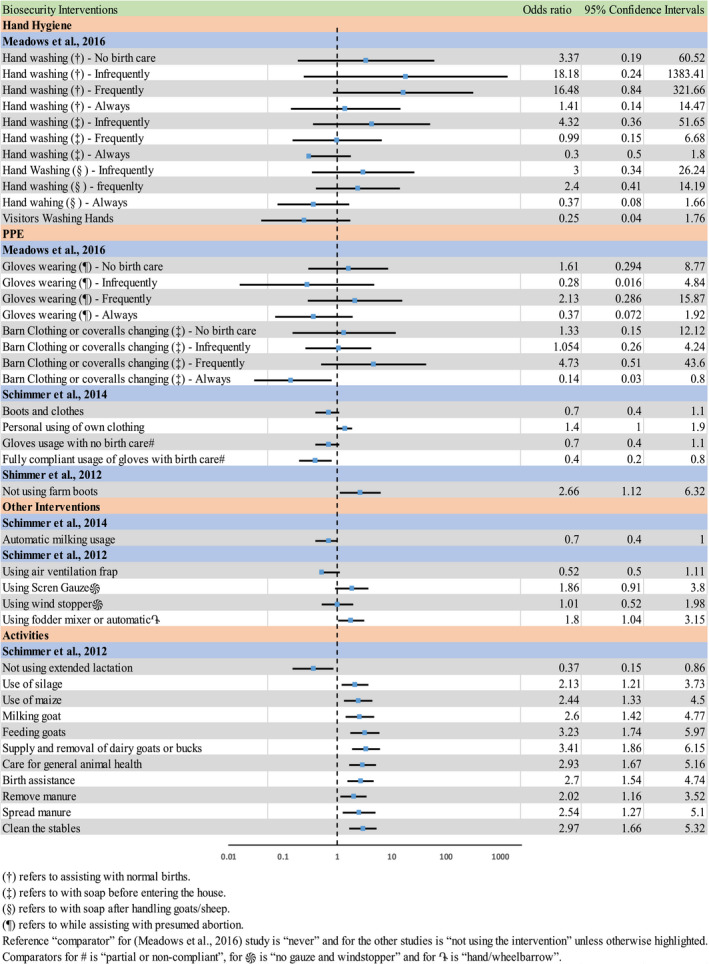
Forest plot of odds ratios related to odds of human colonization or infection with bacteria

Of the studies included in this review, three studies reported results using ORs. These were extracted and visually represented in Figure 4, a forest plot to explore the relationship within and between studies. If multivariate analyses were available, those were chosen above univariate analyses values. One study (discussed in detail in Section 3.4; PPE) presents ORs but based on PPE being an effect modifier, and so results are not directly comparable with the ORs presented in Figure 4; hence, it's exclusion from the plot (Williams et al., 2013). The figure shows many large CIs that indicates some uncertainty and many of these CIs are crossing the threshold of OR = 1. There is less uncertainty in the ‘farm activities’ category; however, all these results come from one study (Schimmer et al., 2012).

### 3.6 Risk of bias across studies

Risk of bias across studies for each outcome can be found in Table 2 (see Appendix S5 Table 5.1 for individual domain scoring). For hand hygiene, sanitization and hygienic measures, the majority of studies were looking at the proportion of colonization and were ‘medium risk’. In general, these studies were scored ‘medium risk’ on the selection bias domain of the checklist. For PPE, the majority of studies were looking at the OR of colonization and were generally scored medium risk of bias. Most of these studies scored ‘high risk’ on the information bias domain and low risk on the ‘confounding’ domain. For the ‘other interventions category,’ the studies were generally medium risk of bias for all of the outcomes measures, in addition to being all scored low in the ‘confounding’ domain. Only one study per type of outcome measure (one proportion‐estimate and one odds‐based estimate) was found in ‘farm activities,’ see Table 2 for their risk of bias.

**TABLE 2:** Summary of included study outcome measure & risk of bias

Study	Setting (Livestock group: Country)	Outcome measure	Overall risk of bias
Hand hygiene, sanitization and hygienic measures			
Racicot et al. (2013)	Poultry: Canada	(Beta coefficient & standard error)	Medium
El‐Tras et al. (2015)	Poultry: Egypt	Proportion colonized	Medium
Meadows et al. (2016)	Sheep and Goats: Canada	OR of colonization	Medium
Abdi et al. (2017)	Poultry: Southern Ethiopia	Proportion colonized	Medium
Ridley et al. (2011)	Broiler: United Kingdom	Proportion colonized	Medium
Leedom Larson et al. (2010)	Pork: United States	Proportion colonized	Medium
Personal protective equipment			
Meadows et al. (2016)	Sheep and Goats: Canada	OR of colonization	Medium
Schimmer et al. (2014)	Dairy Cattle: Netherlands	OR of colonization	Medium
Elmonir et al. (2019)	Dairy cow: Egypt	Proportion colonized	Medium
Williams et al. (2013)	Poultry: England	OR of colonization	High
Schimmer et al. (2012)	Dairy goat: Netherland	OR of colonization	Medium
Vaccination			
Bond et al. (2016)	Goat: Australia	Vaccine efficacy	Medium
Zinsstag et al. (2007)	Cattle and small ruminant: Mongolia	Cost‐effectiveness	
Other interventions			
Schimmer et al. (2014)	Dairy Cattle: Netherland	OR of colonization	Medium
Pletinckx et al. (2013)	Pig: Belgium	Proportion colonized	Medium
Bond et al. (2016)	Goat: Australia	Relative risk of colonization	Medium
Schimmer et al. (2012)	Dairy goat: Netherland	OR of colonization	Medium
Farm activities			
Te‐Chaniyom et al. (2016)	Goat: Thailand	Proportion colonized	Low
Schimmer et al. (2012)	Dairy: Netherland	OR of colonization	Medium

Abbreviation: OR, odds ratio.

## 4 DISCUSSION

### 4.1 Summary of evidence

This review summarizes the effectiveness of biosecurity interventions on farms in relation to the impact on bacterial infection and/or colonization in people. Overall, 14 studies adhered to the inclusion/exclusion criteria and were included in the review. This review collated the types of interventions studied and discussed how they may affect bacterial transmission across One Health through our proposed theory of change. From this, we can see that although most intervention groups focus on the human–animal interface, some also may impact transmission through the environment. Additionally, this review highlights gaps in evidence, through our narrative synthesis approach. For example, we can see a lack of evidence in pig farms for Gram‐negative bacteria and for PPE. Alternatively, there were no hand hygiene studies on cattle‐farms and (across all‐farms) no studies relating to Gram‐positive bacteria aside from *S. aureus*.

The majority of studies were from Europe and the central Asia region, with very little data found from the Middle East or Sub‐Saharan Africa regions. Currently, there is an increased global interest in alternatives to antimicrobials in preventing illness in livestock, with antimicrobials potentially masking the impact of poor hygiene and biosecurity. Europe has strict guidelines on antimicrobial use in livestock production, which may explain why more studies on effectiveness of biosecurity measures have been conducted than elsewhere (Grace, 2015). More global evidence on the effectiveness of biosecurity interventions in terms of human health and productivity will help the international agenda of antibiotic stewardship from a One Health perspective.

Only one economic evaluation study was found (Zinsstag et al., 2007), with such information being crucial for decision‐makers to consider the cost and utility impacts of different biosecurity intervention. More cross‐sectoral intervention evaluations that incorporate both epidemiological and economic impacts are needed to ensure efficient intervention selection (Rüegg et al., 2018).

Many of the results extracted and included in our narrative synthesis are based on univariate analyses with a medium risk of bias. This highlights the need for future studies to consult and adhere to risk of bias assessment checklists (such as those used in this review), and to appropriately utilize statistical techniques in understanding associations between biosecurity interventions and human health outcomes.

#### 4.1.1 Comparison of the literature

This is the first systematic literature review to investigate the effect of biosecurity interventions on human health outcomes. However, our results are aligned with previous literature that is generally related to zoonotic infections. The majority of zoonotic bacterial infections evaluated were *C. burnetii*. This reflects the findings of a review on human–livestock zoonotic infections, where *C. burnetii* was found as a the second major bacterial zoonotic infection (Klous et al., 2016). The studies included in the current review identified the effectiveness of biosecurity interventions on some of the bacterial infections considered by World Health Organization as common and important zoonotic pathogens transmitting from animal to human, (Grace, 2015; World Health Organization, 2019) such as *Salmonella*, *E. coli* and *Campylobacter*. However, there are other important zoonotic bacteria that pose an occupational risk for farm workers for which no evidence was found, such as *Bacillus anthracis* and *Bovine tuberculosis* (Dixon et al., 1999; Morwal & Sharma, 2017; Theon et al., 2006).

Our results show the potential importance of hand washing and hygienic measures as a biosecurity intervention. These results agree with other studies of interventions from an animal perspective where poor cleaning and disinfection was associated with higher risks of poultry flock infections (Cardinale et al., 2004; Johnsen et al., 2006; Newell et al., 2011). Also, results for PPE as biosecurity interventions agreed with other studies where wearing clothes and boots was associated with a risk reduction of infections in poultry flocks (Bouwknegt et al., 2004; Newell et al., 2011). However, more robust evidence is needed before we can recommend such interventions on the basis of human health impact.

### 4.2 Strengths and limitations

This is the first systematic review summarizing and quality assessing studies about the effectiveness of biosecurity interventions on the transmission of bacterial infections from animals to people from a human and occupational hazard perspective at the farm level. In addition, this review used a systematic search protocol, with used databases specializing in both public health and agriculture. Grey literature was not reviewed. However, much of the related grey literature describes general recommendations or intervention types, without actually measuring their effectiveness (European Commission, 2012; Food & Agriculture Organization of the United Nations/United States Agency International Development, 2019; The European Feed Manufacturers' Federation, 2019).

Though a systematic review protocol was used, only around 25% of title/abstract and full articles reviewing was done by the 2nd reviewer, which may add some risk of bias in study selection. However, inter‐rater agreement was above 80% over both the abstract and full‐text stages, indicating overall low selection bias. Given the hypothesized lack of studies that would meet the criteria, the reviewers erred on the side of caution for any subjective cases, reducing the likelihood of wrongly excluded studies at review stage. In addition, studies were not excluded based on quality; however, this was due to the scarcity of studies found about effectiveness of biosecurity interventions.

## 5 CONCLUSIONS

Zoonotic bacterial infections are a serious health risk for people in contact with livestock farms. This review suggests that biosecurity interventions may help in reducing bacterial transmission from livestock to humans, with studies found investigating the impact on hand hygiene, PPE, vaccinations, farm factors and farm activities. However, high‐quality evidence at a global level is needed before strong conclusions can be drawn on which of these intervention types is the most effective for improving human health outcomes, particularly in low‐ and middle‐income countries. Additionally, as the economic evidence is currently lacking, recommendations based on cost‐effectiveness or cost‐benefit results cannot be given. Therefore, we recommend that future studies on biosecurity interventions at the farm level should include human health and economic outcomes in addition to more traditional animal colonization‐based outcomes.

## CONFLICT OF INTEREST

No conflict of interest to declare.

## AUTHOR CONTRIBUTIONS

DY was the lead literature reviewer and developed the study protocol. NN was the second reviewer and helped in developing the protocol. All authors contributed to the drafting of the manuscript, interpretation and communication of the results.

## ETHICAL STATEMENT

This project was formed as part of a MSc Student Project for which an ethical approval was submitted to the LSHTM MSc Research Ethics Committee and assessed by the Research Governance and Integrity Office. This was approved, and given that this was a literature review, based on published evidence, with no primary data collection, no further ethical approval processes were needed.

## Supporting information

Supplementary Material
