# Supplementary material for: The effectiveness of biosecurity interventions in reducing the transmission of bacteria from livestock to humans at the farm level: A systematic literature review
Source: Zoonoses Public Health. 2021 Feb 4;68(6):549–62. doi: 10.1111/zph.12807 (PMC8451914; doi:10.1111/zph.12807)
Supplement: Supplementary file 1 — Supplementary Material [file ZPH-68-549-s001.docx]

**Appendices:**

**Appendix S1:**

Table 1.1. PRISMA Checklist (Moher et al., 2009):

| **Section/topic** | **#** | **Checklist item** | **Reported on page #** |
| --- | --- | --- | --- |
| **TITLE** | | |  |
| Title | 1 | Identify the report as a systematic review, meta-analysis, or both. | P. 1 |
| **ABSTRACT** | | |  |
| Structured summary | 2 | Provide a structured summary including, as applicable: background; objectives; data sources; study eligibility criteria, participants, and interventions; study appraisal and synthesis methods; results; limitations; conclusions and implications of key findings; systematic review registration number. | P. 1 |
| **INTRODUCTION** | | |  |
| Rationale | 3 | Describe the rationale for the review in the context of what is already known. | P. 2 |
| Objectives | 4 | Provide an explicit statement of questions being addressed with reference to participants, interventions, comparisons, outcomes, and study design (PICOS). | P. 2 |
| **METHODS** | | |  |
| Protocol and registration | 5 | Indicate if a review protocol exists, if and where it can be accessed (e.g., Web address), and, if available, provide registration information including registration number. | N/A |
| Eligibility criteria | 6 | Specify study characteristics (e.g., PICOS, length of follow-up) and report characteristics (e.g., years considered, language, publication status) used as criteria for eligibility, giving rationale. | P. 2,3  (Table 1) |
| Information sources | 7 | Describe all information sources (e.g., databases with dates of coverage, contact with study authors to identify additional studies) in the search and date last searched. | P. 2 |
| Search | 8 | Present full electronic search strategy for at least one database, including any limits used, such that it could be repeated. | P. 3  Appendix S2 |
| Study selection | 9 | State the process for selecting studies (i.e., screening, eligibility, included in systematic review, and, if applicable, included in the meta-analysis). | P. 3 |
| Data collection process | 10 | Describe method of data extraction from reports (e.g., piloted forms, independently, in duplicate) and any processes for obtaining and confirming data from investigators. | P. 3,4 |
| Data items | 11 | List and define all variables for which data were sought (e.g., PICOS, funding sources) and any assumptions and simplifications made. | P. 4 |
| Risk of bias in individual studies | 12 | Describe methods used for assessing risk of bias of individual studies (including specification of whether this was done at the study or outcome level), and how this information is to be used in any data synthesis. | P. 4 |
| Summary measures | 13 | State the principal summary measures (e.g., risk ratio, difference in means). | P. 4 |
| Synthesis of results | 14 | Describe the methods of handling data and combining results of studies, if done, including measures of consistency (e.g., I^2^) for each meta-analysis. | P. 4 |

| **Section/topic** | **#** | **Checklist item** | **Reported on page #** |
| --- | --- | --- | --- |
| Risk of bias across studies | 15 | Specify any assessment of risk of bias that may affect the cumulative evidence (e.g., publication bias, selective reporting within studies). | P. 4 |
| Additional analyses | 16 | Describe methods of additional analyses (e.g., sensitivity or subgroup analyses, meta-regression), if done, indicating which were pre-specified. | N/A |
| **RESULTS** | | |  |
| Study selection | 17 | Give numbers of studies screened, assessed for eligibility, and included in the review, with reasons for exclusions at each stage, ideally with a flow diagram. | P. 5  Figure 1 |
| Study characteristics | 18 | For each study, present characteristics for which data were extracted (e.g., study size, PICOS, follow-up period) and provide the citations. | P. 5  Appendix S3 |
| Risk of bias within studies | 19 | Present data on risk of bias of each study and, if available, any outcome level assessment (see item 12). | P. 6  Appendix S5 and S6 |
| Results of individual studies | 20 | For all outcomes considered (benefits or harms), present, for each study: (a) simple summary data for each intervention group (b) effect estimates and confidence intervals, ideally with a forest plot. | P. 6,7  Appendix S4 |
| Synthesis of results | 21 | Present results of each meta-analysis done, including confidence intervals and measures of consistency. | P. 7,8  Figures 2,3,4 |
| Risk of bias across studies | 22 | Present results of any assessment of risk of bias across studies (see Item 15). | P. 8,9  (Table 2) |
| Additional analysis | 23 | Give results of additional analyses, if done (e.g., sensitivity or subgroup analyses, meta-regression [see Item 16]). | N/A |
| **DISCUSSION** | | |  |
| Summary of evidence | 24 | Summarize the main findings including the strength of evidence for each main outcome; consider their relevance to key groups (e.g., healthcare providers, users, and policy makers). | P. 9,11,12 |
| Limitations | 25 | Discuss limitations at study and outcome level (e.g., risk of bias), and at review-level (e.g., incomplete retrieval of identified research, reporting bias). | P. 12 |
| Conclusions | 26 | Provide a general interpretation of the results in the context of other evidence, and implications for future research. | P. 12 |
| **FUNDING** | | |  |
| Funding | 27 | Describe sources of funding for the systematic review and other support (e.g., supply of data); role of funders for the systematic review. | P. 1 |

**Appendix S2:** Search Terms

Appendix S2.1 Search Terms for Ovid Embase Database (Ovid Embase, 2019):

**Search retrieved: 468 studies**

1. biosecurity

2. intervention?

3. method?

4. exp procedures/

5. technique?

6. polic*

7. strateg*

8. exp control strategy/

9. measure?

10. procedure?

11. farm?

12. farm-level

13. livestock

14. exp livestock/

15. exp farm animal/

16. "farm animal?"

17. farmland?

18. farm-land?

19. exp agricultural land/

20. exp agricultural worker/

21. farmer?

22. "agricultur* lab??r*"

23. "agricultr* worker?"

24. farming

25. agrarian

26. "farm worker?"

27. farmworker?

28. effectiveness

29. outcome?

30. infection?, bacteria*

31. exp bacterial infection/

32. "bacterial infection?"

33. "bacteria infection?"

34. "microbial infection?"

35. "bacterium infection?"

36. coloni#ation

37. "bacterial coloni#ation"

38. exp microbial colonization/

39. "microbial coloni#ation"

40. exp bacterial colonization/

41. "bacterium coloni#ation"

42. coloni#ation,bacteria

43. coloni#ation, microbial

44. prevalence?

45. exp prevalence/ or seroprevalence/

46. incidence?

47. "incidence rate"

48. rate,incidence

49. exp "cost effectiveness analysis"/

50. cost-effectiveness

51. "cost effectiveness ratio"

52. analys#s, cost-effectiveness

53. "cost-effectiveness analys#s"

54. "cost-efficiency analys#s"

55. "cost analys#s"

56. effectiveness, cost

57. exp "cost benefit analysis"/

58. "cost benefit"

59. "cost benefit ratio"

60. analys#s, cost-benefit

61. data, cost-benefit

62. analys#s, cost-utility

63. analys#s, marginal

64. "economic evaluation?"

65. evaluation?, economic

66. 2 OR 3 OR 4 OR 5 OR 6 OR 7 OR 8 OR 9 OR 10

67. 11 OR 12 OR 13 OR 14 OR 15 OR 16 OR 17 OR 18 OR 19 OR 20 OR 21 OR 22 OR 23 OR 24 OR 25 OR 26 OR 27

68. 28 OR 29 OR 30 OR 31 OR 32 OR 33 OR 34 OR 35 OR 36 OR 37 OR 38 OR 39 OR 40 OR 41 OR 42 OR 43 OR 44 OR 45 OR 46 OR 47 OR 48 OR 49 OR 50 OR 51 OR 52 OR 53 OR 54 OR 55 OR 56 OR 57 OR 58 OR 59 OR 60 OR 61 OR 62 OR 63 OR 64 OR 65

69. 1 AND 66 AND 67 AND 68

Appendix S2.2 Search Terms for Ovid Medline Database (Ovid Medline, 2019):

**Search retrieved: 367 studies**

1. biosecurity

2. intervention?

3. method?

4. exp procedures/

5. procedure?

6. technique?

7. polic*

8. strateg*

9. exp control strategy/

10. measure?

11. farm?

12. farm-land

13. farmland

14. farm-level

15. exp farm animal/

16. "farm animal?"

17. exp Farms/

18. farmland?

19. farm-land?

20. livestock

21. exp livestock/

22. exp agricultural worker/

23. farmer?

24. "agricultur* lab??r*"

25. "agricultr* worker?"

26. farming

27. agrarian

28. "farm worker?"

29. farmworker?

30. effectiveness

31. outcome?

32. coloni#ation

33. "bacterial coloni#ation"

34. exp microbial colonization/

35. "microbial coloni#ation"

36. exp bacterial colonization/

37. "bacterium coloni#ation"

38. coloni#ation,bacteria

39. coloni#ation, microbial

40. exp bacterial infection/

41. "bacterial infection?"

42. "bacteria infection?"

43. infection?, bacteria*

44. "bacterial infection?"

45. "microbial infection?"

46. "bacterium infection?"

47. "incidence rate"

48. rate,incidence

49. exp incidence/

50. exp prevalence/

51. prevalence?

52. incidence?

53. exp "cost effectiveness analysis"/

54. cost-effectiveness

55. "cost effectiveness ratio"

56. analys#s, cost-effectiveness

57. effectiveness, cost

58. "cost-effectiveness analys#s"

59. "cost-efficiency analys#s"

60. exp "cost benefit analysis"/

61. "cost benefit"

62. "cost benefit ratio"

63. analys#s, cost-benefit

64. data, cost-benefit

65. analys#s, cost-utility

66. analys#s, marginal

67. "economic evaluation?"

68. evaluation?, economic

69. "cost analys#s"

70. 2 OR 3 OR 4 OR 5 OR 6 OR 7 OR 8 OR 9 OR 10

71. 11 OR 12 OR 13 OR 14 OR 15 OR 16 OR 17 OR 18 OR 19 OR 20 OR 21 OR 22 OR 23 OR 24 OR 25 OR 26 OR 27 OR 28 OR 29

72. 30 OR 31 OR 32 OR 33 OR 34 OR 35 OR 36 OR 37 OR 38 OR 39 OR 40 OR 41 OR 42 OR 43 OR 44 OR 45 OR 46 OR 47 OR 48 OR 49 OR 50 OR 51 OR 52 OR 53 OR 54 OR 55 OR 56 OR 57 OR 58 OR 59 OR 60 OR 61 OR 62 OR 63 OR 64 OR 65 OR 66 OR 67 OR 68 OR 69

73. 1 AND 70 AND 71 AND 72

Appendix S2.3 Search Terms for Agris Database (FAO, 2019):

**Search retrieved: 34 studies**

(Biosecurity) AND (Intervention* OR polic* OR measure* OR strateg* OR methods OR procedures OR techniques) AND (Farmer OR farm* OR farm-level OR livestock OR "Farm animal*") AND (Effectiveness OR incidence OR prevalence OR cost-effectiveness OR cost-benefit OR outcome* OR "bacterial infection" OR "bacterial colonization" OR "bacterial colonisation" OR colonization OR colonisation OR "microbial colonization" OR "microbial colonisation" OR "cost-effectiveness analysis" OR "cost-effectiveness ratio" OR "cost-benefit ratio" OR "cost-benefit analysis" OR "cost analysis")

**Appendix S3:**

Table 3.1. Studies’ Characteristics:

| **Study** | **Study Title** | **Study Aim/Objective** |
| --- | --- | --- |
| Racicot et al., 2013 | Assessing most practical and effective protocols to sanitize hands of poultry catching crew members. | To assess the effectiveness of practical hand sanitization of four protocols and to identify the most practical one. |
| El-Tras et al., 2015 | Campylobacter infections in children exposed to infected backyard poultry in Egypt. | To make a comparison of the risk of *Campylobacter* faecal carriage between children exposed to *Campylobacter*-infected backyard poultry and those children exposed to non-infected backyard and to evaluate the possible risk factors for a poultry backyard being classified as infected. |
| Meadows et al., 2016 | *Coxiella burnetii* (Q Fever) Seropositivity and Associated Risk Factors in Sheep and Goat Farm Workers in Ontario, Canada. | To evaluate the *Coxiella burnetii (C. burnetii)* seroprevalence among farm workers who care for goats and sheep on Ontario, Canada, also to identify farm management practices, lifestyle measures and demographic characteristics for association with C. burnetii exposure in this population. |
| Schimmer et al., 2014 | *Coxiella burnetii* seroprevalence and risk for humans on dairy cattle farms, the Netherlands, 2010-2011. | To explore the seroprevalence of *C. burnetii* antibodies in workers and residents of dairy cattle farm and find out the participant based and farm-based risk factors for seropositivity. |
| Abdi et al., 2017 | Determination of the sources and antimicrobial resistance patterns of *Salmonella* isolated from the poultry industry in Southern Ethiopia. | To identify poor farm managements in biosecurity that help in pathogen flare-ups, determine the prevalence and find out antimicrobial resistance patterns in distribution and multiplication centres in southern Ethiopia and in three poultry breeding centres. |
| Elmonir et al., 2019 | Ecology of *Staphylococcus aureus* and its antibiotic resistance genes in dairy farms: Contributing factors and public health implications. | To identify important sources and factors related to *Staphylococcus aureus (S. aureus)* dissemination and its resistance genes in dairy farms in Egypt, also, farm workers Knowledge, attitudes and practices were assessed. |
| Pletinckx et al., 2013 | Evidence of possible methicillin-resistant *Staphylococcus aureus* ST398 spread between pigs and other animals and people residing on the same farm. | To enhance the understanding of the possible transmission pathways between animal species, human and barn environment on the same farm through: 1) determining *methicillin-resistant Staphylococcus aureus* (MRSA) prevalence, 2) comparing the resulting spa-, SCCmec types and antibiotics profiles in various animal species, animal workers and barn environment, 3) identify whether *MRSA* carriage in pigs is age-related, 4) assess whether there is a correlation between MRSA-positive sows and suckling piglets. |
| Te-Chaniyom et al., 2016 | Goat farm management and *Brucella* serological test among goat keepers and livestock officers, 2011-2012, Nakhon Si Thammarat Province, southern Thailand. | To evaluate the possible risk factors by type of goat farm and to identify the prevalence of human *Brucella* seropositivity among livestock officer and goat keepers in Nakhon Si Thammarat, Thailand. |
| Zinsstag et al., 2007 | Human Benefits of Animal Interventions for Zoonosis Control. | To demonstrate the conditions for which control of zoonosis would save money for limited-resource countries and likely decrease zoonosis occurrence worldwide. For brucellosis, the aim is to estimate the mass brucellosis vaccination of cattle and small ruminants economic benefit, cost-effectiveness, and distribution of benefit (to public health, agricultural sector and society). |
| Bond et al., 2016 | One Health approach to controlling a Q fever outbreak on an Australian goat farm. | To describe the outbreak of Q fever, the relation to intensive goat farming and the one health management approach. |
| Ridley et al., 2011 | Potential sources of *Campylobacter* infection on chicken farms: Contamination and control of broiler-harvesting equipment, vehicles and personnel. | To assess the effectiveness of additional biosecurity measures on farm aimed at decreasing campylobacter contamination of personnel, vehicles and broiler-harvesting equipment. Also, to evaluate the risk of flock infection at slaughter house from insufficient cleaning of bird transport crates. |
| Williams et al., 2013 | Risk exposures for human ornithosis in a poultry processing plant modified by use of personal protective equipment: an analytical outbreak study. | To describe an analytical study for outbreak in order to evaluate risk factors and to identify control measures beyond the case findings and an initially developed education. |
| Leedom Larson et al., 2010 | Self-reported methicillin-resistant *Staphylococcus aureus* infection in USA pork producers. | To check the reports of skin infection or soft tissue infection in pigs and workers of USA pork producers and to evaluate possible risk factors for the infection related to farm biosecurity such as clothing, shower, laundry and hygiene measures. |
| Schimmer et al., 2012 | Seroprevalence and risk factors for *Coxiella burnetii* (Q fever) seropositivity in dairy goat farmers' households in the Netherlands, 2009-2010. | To determine the seroprevalence in farmers and household members living and/or working on dairy goat farms within the same farm study, and to evaluate risk factors for seropositivity that is related to farm and individual aspects to make control interventions up-to-date and to give tailored advice for this occupational group and the Dutch dairy goat industry. |

**Appendix S4:**

Table 4.1. Studies’ Results

| **Study** | **Study Design** | **Type of Infection** | **Outcome Measures** | **Population** | **Location** | **Type of Intervention** | **Results** |
| --- | --- | --- | --- | --- | --- | --- | --- |
| Racicot et al., 2013 | Pre – post experimental study | *Salmonella* bacteria, *Escherichia coli* and coliforms population | - For number of coliforms and total aerobics: Mean (Standard deviation) (in log) depending on the protocol across the four crew members - For before and after applying the protocol: the mean difference (in log and in percentage) . | Poultry catching crew members | Canada | **Hand sanitization in 4 protocols:**  1) Gel contains alcohol (62% ethanol) 2) Water with triclosan antibacterial soap 3) Degreasing pumice cream and hand wipes  4) Antimicrobial wipes | **For *Salmonella*:**   - All washed hands were negative with each protocol.   **For mean Coliform Count:**   - On mean total coliform count, all protocols had statistically significant effect (P= 0.04). - On initial hand contamination, all protocols had positive effect (*Beta coefficient* [SE]: 0.23 [0.086], P < 0.0001). - Only statistically significant difference between protocol at high initial contamination level where protocol 1 had higher mean contamination level in clean hand than protocol 3 (P= 0.002).   **For total Aerobic bacterial counts:**   - On mean total aerobic bacterial counts, no statistically significant effect of any protocol (P= 0.15). - On initial hand contamination, all protocols had positive effect (*B* [SE]: 0.54 [0.24], P < 0.0001). - Only statistically significant difference between protocols at median (P= 0.002) and high (P= 0.001) initial contamination level where protocol 4 had higher mean contamination level in clean hand than protocol 2. |
| El-Tras et al., 2015 | Cross sectional | *Campylobacter* | Prevalence of *Campylobacter* infection in household | Poultry and farm residents | Egypt | Cleaning and disinfection | **Household *Campylobacter coli* status with poor cleaning and disinfection:**   - Negative *Campylobacter coli* was estimated to have a prevalence (53.6%) - Positive *Campylobacter coli* was estimated to have a prevalence (80%)   **Household *Campylobacter jejuni* *(C. jejuni)* status with poor cleaning and disinfection:**   - Negative *C. jejuni* was estimated to have a prevalence (37%) - Positive *C. jejuni* was estimated to have a prevalence (79%)   (Household means both poultry and children). |
| Meadows et al., 2016 | Cross-Sectional | *Coxiella burnetii (C. burnetii)* | Odds ratio of *C. burnetii* seropositivity in farm workers | Sheep and goat farms and farm workers | Canada | - Gloves - Hand washing - Clothes changing | **Univariable mixed-effects logistic model:**  **Gloves wearing while assisting with presumed abortion in each of the following compared to never wearing gloves:**   - Not applicable (No birth assisting); OR 1.61, 95%CI (0.294–8.77), P= 0.59 - Infrequently; OR 0.28, 95%CI (0.016–4.84), P= 0.38 - Frequently; OR 2.13, 95%CI (0.286–15.87), P= 0.46 - Always; OR 0.37, 95%CI (0.072–1.92), P= 0.024   **Hand washing after assisting with normal births in each of the following compared to never washing hands:**   - Not applicable (No birth assisting); OR 3.37, 95%CI (0.19–60.52), P= 0.041 - Infrequently; OR 18.18, 95%CI (0.24–1383.41), P= 0.19 - Frequently; OR 16.48, 95%CI (0.84–321.66), P= 0.065 - Always; OR 1.41, 95%CI (0.14–14.47), P= 0.77   **Hand washing with soap after handling goats/sheep in each of the following compared to never washing hands:**   - Infrequently; OR 4.32, 95%CI (0.36–51.65), P= 0.25 - Frequently; OR 0.99, 95%CI (0.15–6.68), P= 0.99 - Always; OR 0.30, 95%CI (0.05–1.80), P= 0.19   **Hand washing with soap before entering the house in each of the following compared to never washing hands:**   - Infrequently; OR 3.00, 95% CI (0.34–26.24), P= 0.32 - Frequently; OR 2.40, 95%CI (0.41–14.19), P= 0.33 - Always; OR 0.37, 95%CI (0.08–1.66), P= 0.19   **Barn clothing or coveralls changing after assisting with normal births in each of the following compared to never changing clothes:**   - Not applicable (No birth assisting); OR 1.33, 95%CI (0.15–12.12), P= 0.80 - Infrequently; OR 1.054, 95%CI (0.26–4.24), P= 0.94 - Frequently; OR 4.73, 95%CI (0.51–43.60), P= 0.017 - Always; OR 0.14, 95%CI (0.03–0.80), P= 0.027   Visitors hand washing; OR 0.25, 95%CI (0.04–1.76), P= 0.16 |
| Schimmer et al., 2014 | Cross sectional | *C. burnetii* | Odds ratio of *C. burnetii* positivity among dairy cattle farm  Residents | Dairy cattle and  farm residents, farm workers and professionals | Netherlands | - Personal Protective Equipment (PPE) such as: Gloves Boots   Farm clothes   - Automatic milking | **Univariate logistic model:**   - Professional visitors Boots and clothes; OR 0.7, 95%CI (0.4–1.1). Own personnel clothes; OR 1.4, 95%CI (1.0–1.9).   **Multivariate logistic regression analysis:**   - Fully compliant gloves usage; OR 0.4, 95%CI (0.2–0.8). - No birth care; OR 0.7, 95% CI (0.4–1.1). - Automatic milking usage; OR 0.7, 95%CI (0.4–1.0). |
| Abdi et al., 2017 | Cross sectional | *Salmonella* | Prevalence of *Salmonella* in farm attendants’ hand swab | Poultry  Farm workers | Southern Ethiopia | - Hand washing | - Farm attendants' hand swab carry *Salmonella*= 3/9 (33%) - Farm attendants in all study areas weren’t washing their hands before coming in contact with chicken. |
| Elmonir et al., 2019 | Cross sectional | *Staphylococcus aureus (S. aureus)* | Prevalence of *S. aureus* in farm workers’ nostril and hand swap | Cow dairy farm  farm workers | Egypt | - PPE | - Farm workers had overall prevalence 88.9% of *S. aureus* in nostril swap and 100% in hand swab. - All farm workers were not using PPE. |
| Pletinckx et al., 2013 | Cross sectional | Methicillin-resistant *Staphylococcus aureus* (MRSA) | Prevalence of MRSA in farmers, family members and pig veterinaries. | - Farm A, B are pig farms - Farms C, D are mixed broiler-pig, farms E and F are mixed dairy-pig farms. - Farmers, family members and pig veterinarians. | Belgium | - Isolated building with separate air supply | - Farmers, family members and pig veterinaries have 0% prevalence of MRSA in farm B. - Farm B was the only one using separate building with separate air supply. |
| Te-Chaniyom et al., 2016) | Cross sectional | *Brucella* | Prevalence of *Brucella* seropositivity in livestock officers | Goat farm and livestock officers | Thailand | Activities such as:   - Conducting vaccination - Contacting placenta and vaginal secretions - Blood collection - Artificial insemination | - Prevalence of livestock officers tested positive for *Brucella*= 3/34 (8.8%), 95%CI (1.9%-23.7%). There was no significant association between any of these activities and officers seropositivity. |
| Zinsstag et al., 2007 | Economic modelling | *Brucella* | Cost effectiveness ratio and Disability-adjusted life years (DALY) of the intervention | Cattle and small ruminants farms | Mongolia | - *Brucella melitensis* Rev-1 for small ruminants and *Brucella abortus* S19 for cattle as annual mass vaccination for 10 year | **By decreasing the brucellosis transmission between animals by 52%:**   - 51,856 human brucellosis cases could be avoided - 49,207 DALYS could be avoided with US$26.6 million economic benefit, and at a cost of US$ 8.3 million, cost effectiveness ratio of US$19.1 per DALY averted (US$ in year 2000) (95% CI 5.3–486.8) - By allocating costs of vaccination proportionally to all benefit, for agricultural and public health sectors, the intervention is cost effective and cost saving. |
| Bond et al., 2016 | Retrospective cohort | *C. burnetii* | - Relative risk of *C. burnetii* infection - Vaccine efficacy | Goat farm and  farm workers | Australia | - High efficiency particulate arrestance (HEPA) filter - Human Q fever vaccine (Q-Vax®, CSL Ltd, Australia) | **HEPA Filter Relative Risk (CI):**   - The risk of *C. burnetii* infections among Administrative staff in unfiltered adjoining office is 5.49 (RR 5.49, 95%CI 1.29-23.4) and among workers regularly handling goat and kids is 5.56 (RR 5.56, 95%CI 1.09-29.3) times the risk of infection among workers in a HEPA filter factory.   **Q fever human vaccine**   - Farm workers vaccinated 15 days before exposure had vaccine efficacy >90% which was a way of preventing further cases of acute Q fever. |
| Ridley et al., 2011 | pre-post intervention | *Campylobacter* | Prevalence of *Campylobacter* positive samples | Broiler and catching crew | United Kingdom | - Sanitization and hand washing - Personal protective equipment such as clean clothing and footwear disinfected with a 1 ⁄ 120 dilution of Virkon S (DuPont, Sudbury, UK) | - *Campylobacter* positive samples prevalence decreased from 41-19% in footwear and from 14%-10% in hand after footwear and hand cleaning, at (P= 0.002). |
| Williams et al., 2013 | Case-control | *Chlamydia psittaci* | Odds ratio of *Chlamydia psittaci* infection | Poultry and farm staff | England | Personal Protective Equipment:   - Eye protection - Respiratory mask | **Self-infection through touching face with contaminated hands:**  Measured through modified effect (stratified by PPE):   - Staff using eye protection sometimes or always has OR 0.75, 95%CI (0.01–19.9) and never used OR 31, 95%CI (2.04–1572), p=0.04 - Staff using respiratory protection FFP3 mask has OR 1.2, 95%CI (0.02–19.3) and never used OR= infinity, 95%CI (4.8-infinity), P= 0.03   **Worked in or visited automated evisceration or killing areas:**  Measured through modified effect (stratified by PPE):   - Staff using eye protection sometimes or always has OR=infinity, 95%CI (0.38–infinity) and never used OR 6, 95%CI (0.71–71.8), P= 0.56 - Staff using respiratory protection FFP3 mask has OR 1.6, 95%CI (0.1–94.3) and never used OR 30, 95%CI (1.3–1647), P= 0.11 |
| Leedom Larson et al., 2010 | Cross-sectional | MRSA | Prevalence of MRSA infection | Pork Producers | United States | - Work Laundry separation | - MRSA infection in workers: 5/135 (3.7%) of workers has a history of MRSA skin infection or soft tissue infection Separating work laundry from other types as a possible risk factor for MRSA infection in workers is not significant P= 0.11. |
| Schimmer et al., 2012 | Cross sectional | *C. burnetii* | Odds ratio of *C. burnetii* infection | Dairy goat and farmers | Netherland | Interventions:   - Boots - Air ventilation frap - Not using extended lactation - Feeding method - Using silage - Using maize - Using screen/gauze - Feeding and milking goats - Animal health care - Remove and spread manure - Birth assistance - Stable cleaning | **Univariate multilevel model:**   - Not using extended lactation; OR 0.43 (95%CI 0.20-0.91) P= 0.03, compared to using extended lactation - Using air ventilation flap; OR 0.52, 95%CI (0.5-1.11), P= 0.10, compared to not using - Using fodder mixer/automatic instead of hand/wheelbarrow for feeding method; OR 1.8, 95%CI (1.04-3.15), P= 0.04. - Not using farm boots; OR 1.93, 95%CI (1.03-3.06), P= 0.03, compared to using farm boots. - Use of silage; OR 2.13, 95%CI (1.21–3.73), P< 0.01, compared to no use of silage - Use of maize; OR 2.44, 95%CI (1.33–4.50), P< 0.01, compared to no use of maize - Use of screen/gauze; OR 1.86, 95%CI (0.91–3.80), P= 0.12, Windstoppers only; OR 1.01, 95%CI (0.52–1.98) - Milking goat; OR 2.60, 95%CI (1.42–4.77), P< 0.01, compared to not milking goats - Feeding goats; OR 3.23, 95%CI (1.74–5.97), P< 0.01, compared to not feeding goats - Supply and removal of dairy goats or bucks; OR 3.41, 95%CI (1.89–6.15), P< 0.01, compared to no supply and removal of dairy goats or bucks - Care for general animal health; OR 2.93, 95%CI (1.67–5.16), P< 0.01, compared to no care for general animal health - Birth assistance; OR 2.70, 95%CI (1.54–4.74), P< 0.01, compared to no birth assistance - Remove manure; OR 2.02, 95%CI (1.16–3.52), P= 0.01, compared to not removing manure - Spread manure; OR 2.54, 95%CI (1.27–5.10), P< 0.01, compared to not spreading manure - Clean the stables; OR 2.97, 95% (1.66–5.32), P< 0.01, compared to not cleaning stables   **Multilevel analysis:**   - Not using extended lactation; OR 0.37, 95%CI (0.15-0.86), P= 0.036, compared to using extended lactation - Not using farm boots; OR 2.66, 95%CI (1.12-6.32), P= 0.025, compared to using farm boots |

p, p-value; CI, Confidence Interval; SE, standard error; OR, odds ratio; RR, relative risk.

**Appendix S5:**

Table 5.1: Overall Risk of Bias and Individual Assessment Criteria Results

| Cross-Sectional Studies | | | | |
| --- | --- | --- | --- | --- |
| **Study** | **Selection Bias** | **Information Bias (Measurement bias)** | **Confounding** | **Risk of bias** |
| El-Tras et al., 2015 | Medium | Medium | High | Medium |
| Meadows et al., 2016 | Low | High | Low | Medium |
| Schimmer et al., 2014 | Medium | High | Low | Medium |
| Abdi et al., 2017 | Medium | Medium | Low | Medium |
| Elmonir et al., 2019 | Medium | Medium | High | Medium |
| Pletinckx et al., 2013 | Medium | High | Low | Medium |
| Te-Chaniyom et al., 2016 | Low | Low | Low | Low |
| Leedom Larson et al., 2010 | Medium | High | Low | Medium |
| Schimmer et al., 2012 | Medium | Low | Low | Medium |
| Cohort Studies | | | | |
| **Study** | **Selection Bias** | **Information Bias (Measurement Bias)** | **Confounding** | **Risk of bias** |
| Bond et al., 2016 | Low | High | Low | Medium |
| Case-Control Studies | | | | |
| **Study** | **Selection Bias** | **Information Bias (Measurement Bias)** | **Confounding** | **Risk of Bias** |
| Williams et al., 2013 | High | High | Low | High |
| Pre-Post Studies | | | | |
| **Study** | **Selection Bias** | **Information Bias (Measurement Bias)** | **Confounding** | **Risk of bias** |
| Racicot et al., 2013 | High | Low | Low | Medium |
| Ridley et al., 2011 | Medium | Low | Low | Medium |

**Appendix S6:** Risk of Bias Assessment Checklists

Appendix S6.1: National Institute of Health Tool, Quality Assessment for Cross-sectional Studies (National Heart, Lung and Blood Institute, 2019):

| **Study Identification** | **Q1** | **Q2** | **Q3** | **Q4** | **Q5** | **Q6** | **Q7** | **Q8** | **Q9** | **Q10** | **Q11** | **Q12** | **Q13** | **Q14** |
| --- | --- | --- | --- | --- | --- | --- | --- | --- | --- | --- | --- | --- | --- | --- |
| El-Tras et al., 2015 | YES | YES | N/R† | YES | YES | NO | NO | N/A‡ | YES | N/A | YES | N/A‡ | N/A‡ | NO |
| Meadows et al., 2016 | YES | YES | YES | YES | NO | NO | NO | YES | NO | N/A | YES | N/A‡ | N/A‡ | YES |
| Schimmer et al., 2014 | YES | YES | NO | YES | YES | NO | NO | YES | NO | N/A | YES | N/A‡ | N/A‡ | YES |
| Abdi et al., 2017 | YES | YES | N/R† | YES | YES | NO | NO | N/A‡ | NO | N/A | YES | N/A‡ | N/A‡ | YES |
| Elmonir et al., 2019 | YES | YES | N/R† | YES | NO | NO | NO | YES | YES | N/A | YES | N/A‡ | N/A‡ | NO |
| Pletinckx et al., 2013 | YES | YES | N/R† | YES | YES | NO | NO | N/A‡ | NO | N/A | YES | N/A‡ | N/A‡ | N/A‡ |
| Te-Chaniyom et al., 2016 | YES | YES | YES | YES | YES | NO | NO | N/A‡ | YES | N/A | YES | N/A‡ | N/A‡ | YES |
| Leedom Larson et al., 2010 | YES | YES | NO | YES | NO | NO | NO | N/A‡ | NO | N/A | NO | N/A‡ | N/A‡ | YES |
| Schimmer et al., 2012 | YES | YES | NO | YES | NO | NO | NO | YES | YES | N/A | YES | N/A‡ | N/A‡ | YES |

†N/R, Not reported.

‡N/A, Not applicable.

Appendix S6.2 National Institute of Health Tool, Quality Assessment for Cohort Studies (National Heart, Lung and Blood Institute, 2019):

| **Study Identification** | **Q1** | **Q2** | **Q3** | **Q4** | **Q5** | **A6** | **Q7** | **Q8** | **Q9** | **Q10** | **Q11** | **Q12** | **Q13** | **Q14** |
| --- | --- | --- | --- | --- | --- | --- | --- | --- | --- | --- | --- | --- | --- | --- |
| Bond et al., 2016 | YES | YES | YES | YES | NO | YES | N/R† | NO | NO | NO | YES | N/R† | N/A‡ | YES |

†N/R, Not reported.

‡N/A, Not applicable.

Checklist questions are same for cross-sectional and cohort studies as following:

Q1. Was the research question or objective in this paper clearly stated?

Q2. Was the study population clearly specified and defined?

Q3. Was the participation rate of eligible persons at least 50%?

Q4. Were all the subjects selected or recruited from the same or similar populations (including the same time period)? Were inclusion and exclusion criteria for being in the study prespecified and applied uniformly to all participants?

Q5. Was a sample size justification, power description, or variance and effect estimates provided?

Q6. For the analyses in this paper, were the exposure(s) of interest measured prior to the outcome(s) being measured?

Q7. Was the timeframe sufficient so that one could reasonably expect to see an association between exposure and outcome if it existed?

Q8. For exposures that can vary in amount or level, did the study examine different levels of the exposure as related to the outcome (e.g. categories of exposure, or exposure measured as continuous variable)?

Q9. Were the exposure measures (independent variables) clearly defined, valid, reliable, and implemented consistently across all study participants?

Q10. Was the exposure(s) assessed more than once over time?

Q11. Were the outcome measures (dependent variables) clearly defined, valid, reliable, and implemented consistently across all study participants?

Q12. Were the outcome assessors blinded to the exposure status of participants?

Q13. Was loss to follow-up after baseline 20% or less?

Q14. Were key potential confounding variables measured and adjusted statistically for their impact on the relationship between exposure(s) and outcome(s)?

Appendix S6.3 National Institute of Health Tool, Quality Assessment of Case-control Studies (National Heart, Lung and Blood Institute, 2019):

| **Study Identification** | **Q1** | **Q2** | **Q3** | **Q4** | **Q5** | **A6** | **Q7** | **Q8** | **Q9** | **Q10** | **Q11** | **Q12** |
| --- | --- | --- | --- | --- | --- | --- | --- | --- | --- | --- | --- | --- |
| Williams et al., 2013 | YES | YES | N/R | NO | NO | YES | NO | NO | YES | NO | †N/R | YES |

†N/R, Not reported

Checklist questions for case-control studies:

Q1. Was the research question or objective in this paper clearly stated and appropriate?

Q2. Was the study population clearly specified and defined?

Q3. Did the authors include a sample size justification?

Q4. Were controls selected or recruited from the same or similar population that gave rise to the cases (including the same timeframe)?

Q5. Were the definitions, inclusion and exclusion criteria, algorithms or processes used to identify or select cases and controls valid, reliable, and implemented consistently across all study participants?

Q6. Were the cases clearly defined and differentiated from controls?

Q7. If less than 100 percent of eligible cases and/or controls were selected for the study, were the cases and/or controls randomly selected from those eligible?

Q8. Was there use of concurrent controls?

Q9. Were the investigators able to confirm that the exposure/risk occurred prior to the development of the condition or event that defined a participant as a case?

Q10. Were the measures of exposure/risk clearly defined, valid, reliable, and implemented consistently (including the same time period) across all study participants?

Q11. Were the assessors of exposure/risk blinded to the case or control status of participants?

Q12. Were key potential confounding variables measured and adjusted statistically in the analyses? If matching was used, did the investigators account for matching during study analysis?

Appendix S6.4 National Institute of Health Tool, Quality Assessment of Pre-Post Intervention Studies (National Heart, Lung and Blood Institute, 2019):

| **Study Identification** | **Q1** | **Q2** | **Q3** | **Q4** | **Q5** | **Q6** | **Q7** | **Q8** | **Q9** | **Q10** | **Q11** | **Q12** |
| --- | --- | --- | --- | --- | --- | --- | --- | --- | --- | --- | --- | --- |
| Racicot et al., 2013 | YES | YES | NO | NO | YES | YES | YES | N/A‡ | YES | YES | YES | N/A‡ |
| Ridley et al., 2011 | YES | NO | YES | NO | NO | YES | YES | N/A‡ | YES | YES | YES | N/R† |

†N/R, Not reported.

‡N/A, Not applicable.

Checklist questions for pre-post studies:

Q1. Was the study question or objective clearly stated?

Q2. Were eligibility/selection criteria for the study population prespecified and clearly described?

Q3. Were the participants in the study representative of those who would be eligible for the test/service/intervention in the general or clinical population of interest?

Q4. Were all eligible participants that met the prespecified entry criteria enrolled?

Q5. Was the sample size sufficiently large to provide confidence in the findings?

Q6. Was the test/service/intervention clearly described and delivered consistently across the study population?

Q7. Were the outcome measures prespecified, clearly defined, valid, reliable, and assessed consistently across all study participants?

Q8. Were the people assessing the outcomes blinded to the participants' exposures/interventions?

Q9. Was the loss to follow-up after baseline 20% or less? Were those lost to follow-up accounted for in the analysis?

Q10. Did the statistical methods examine changes in outcome measures from before to after the intervention? Were statistical tests done that provided p values for the pre-to-post changes?

Q11. Were outcome measures of interest taken multiple times before the intervention and multiple times after the intervention (i.e. did they use an interrupted time-series design)?

Q12. If the intervention was conducted at a group level (e.g. a whole hospital, a community) did the statistical analysis take into account the use of individual-level data to determine effects at the group level?

Appendix S6.5 CASP Tool, Economic Evaluation Study (Critical Appraisal Skills Programme, 2018):

| **Study Identification** | **Q1** | **Q2** | **Q3** | **Q4** | **Q5** | **Q6** | **Q7** | **Q8** | **Q9** | **Q10** | **Q11** | **Q12** |
| --- | --- | --- | --- | --- | --- | --- | --- | --- | --- | --- | --- | --- |
| Zinsstag et al., 2007 | YES | YES | YES | YES | YES | YES | YES | YES | YES | YES | YES | YES |

Checklist questions for economic evaluation studies:

**(I) Is the economic evaluation valid?**

Q1. Was a well-defined question posed?

Q2. Was a comprehensive description of the competing alternatives given?

Q3. Does the paper provide evidence that the programme would be effective? (i.e. would the programme do more good than harm?)

Q4. Were the effects of the intervention identified, measured and valued appropriately?

**(II) How were consequences and costs assessed and compared?**

Q5. Were all important and relevant resources required, and health outcome costs for each alternative identified, measured in appropriate units and valued credibly?

Q6. Were costs and consequences adjusted for different times at which they occurred (discounting)?

Q7. What were the results of the evaluation?

Q8. Was an incremental analysis of the consequences and cost of alternatives performed?

Q9. Was an adequate sensitivity analysis performed?

**(III) Will the results help in purchasing for local people?**

Q10. Is the programme likely to be equally effective in your context or setting?

Q11. Are the costs translatable to your setting?

Q12. Is it worth doing in your setting?
